# Exploring Pathways from Childhood Adversity to Substance Use in Young Adults

**DOI:** 10.3390/ijerph22111608

**Published:** 2025-10-22

**Authors:** Liudas Vincentas Sinkevicius, Sandra Sakalauskaite, Mykolas Simas Poskus, Rasa Pilkauskaite Valickiene, Danielius Serapinas

**Affiliations:** 1 Institute of Psychology, Mykolas Romeris University, 08303 Vilnius, Lithuania; 2 Department of Health Psychology, Lithuanian University of Health Sciences, 47181 Kaunas, Lithuania; 3 Laboratory of Immunology, Department of Immunology and Allergology, Lithuanian University of Health Sciences, 50161 Kaunas, Lithuania; 4 Department of Family Medicine, Medical Academy, Lithuanian University of Health Sciences, 50161 Kaunas, Lithuania

**Keywords:** adverse childhood experiences, alcohol, inflammation, internal and external stress, marijuana, nicotine, substance abuse, substance use, SUD

## Abstract

Adverse childhood experiences (ACEs) are recognized risk factors for later substance use. Yet, data remain scarce—particularly regarding the differentiated effects of specific types of ACEs and their distinct associations with various psychoactive substances. The current study is one of the first in Lithuania to explore the associations between specific ACEs and psychoactive substance use in young adulthood (ages 18–29). This cross-sectional study included a total of 709 participants who completed an online survey. ACEs were measured using a combination of adapted ACEs items and the MACE questionnaire. Substance use was assessed using self-reported instruments: CUDIT-R (cannabis), AUDIT (alcohol), ASSIST (heavy psychoactive substances), and nicotine use. Structural equation modeling (SEM) was chosen to examine predictive relationships. Results revealed that experiences of sexual abuse and physical maltreatment in childhood predicted higher levels of alcohol use in young adulthood. Sexual abuse was positively associated with nicotine, cannabis, and heavy psychoactive substance use, while witnessing interpersonal violence was only associated with higher nicotine use. However, verbal abuse showed significant negative associations across several substance categories. No significant associations were found between family addiction history and substance use. The absence of an important relationship between family history of addiction and substance use indicates that genetic factors may be less decisive than environmental or psychosocial conditions. The main findings of this study are that ACEs are not qualitatively equivalent to one another, so it is worth examining them separately, rather than summing them. Furthermore, based on the negative associations with verbal abuse and the generally statistically negative associations, we can assume that ACEs may not be the most important factors increasing substance use. Further studies should look for other factors that influence substance use.

## 1. Introduction

Adverse childhood experiences (ACEs) are potentially traumatic events occurring in the first eighteen years of life, including physical, emotional, and sexual abuse, neglect, and various family dysfunctions. Adolescence is an intermediate link between early stress and later addictions [1]. The picture of addiction formation is large and influenced by various factors, such as genetic and biological factors, but also the social environment, parental and educational context, drug availability, community background, legislation, policy, perceived substance use norms, or even social media influence serve as recognized risk factors for addiction [2,3,4,5]. ACEs are considered to be a significant risk factor potentially having a long-term impact on a person’s mental and physical health [6]. ACEs are associated with neurophysiological changes in brain development that make individuals more sensitive to stress. Other approaches, such as moral theories, explain addiction as a consequence of moral weakness or spiritual deficiency. However, there are more empirically-based models, such as biological ones. This direction emphasizes genetic and neurobiological factors, claiming that specific biological differences, such as neurotransmitter imbalances or brain structure, can increase the tendency to addiction. Recent research supports a mechanism by which substance use acts as a catalyst to increase sensitivity to stressful life situations through neurobiological changes, particularly by disrupting the stress system and increasing the sensitivity of the reward system [7]. The neuroimmune network hypothesis [8] proposes that ACEs affect the corticoamygdala circuitry, increasing vigilance and threat reactions, and enhancing the stress response via the sympathetic nervous system and the hypothalamic–pituitary–adrenal (HPA) axis. This suggests that early life stress may induce a common neurobiological substrate, particularly involving HPA axis dysregulation and the corticolimbic circuit, which underlies increased stress and increased propensity to use psychoactive substances later in development.

The aforementioned effects can lead to the development of long-term stress sensitivity. To explain these complex processes, we can refer to the multilevel adaptive stress response model presented by R. Sinhna. A normal stress response has three phases: (i) a state of calm, (ii) an active response, and (iii) recovery, which is returning to homeostasis. However, early stress, trauma, and ACEs cause long-term HPA axis hyperactivity, autonomic system imbalance, and impaired prefrontal cortex and limbic system interaction. These changes result in increased stress sensitivity, reduced self-regulation capacity, and a greater tendency to use substances as a compensatory mechanism. At the same time, the author emphasizes the reverse process—the use of psychoactive substances itself disrupts stress regulation: initially stimulating the HPA axis and sympathetic activity, but eventually leading to blunted stress reactivity (weakened cortisol and autonomic responses), emotional imbalance, increased cravings, and risk of relapse. This creates a cycle of stress and substance use in which both directions reinforce each other [9]. Therefore, early experiences may not only lead to altered stress regulation mechanisms, lower resilience to stress, but also to increased susceptibility to risky behaviors [10]. Additionally, ACEs are linked to chronic, low-grade inflammation caused by glucocorticoid insensitivity. Studies show that subjects that experienced higher number of ACEs have higher levels of inflammatory biomarkers [11,12], while inflammation is associated with both mental and physical health problems. The threat response system and immune mechanisms interact with each other, forming a positive feedback loop that can maintain both inflammation and stress sensitivity throughout life [8]. Therefore, ACEs represent early and often chronic stressors, which lead to biological and behavioral dysregulation, and the sensitization to stress is one of the common possible mechanisms through which childhood adversity may confer risk for psychopathology [13].

Epidemiological studies show that increasing levels of substance use disorders (SUDs) (the prevalence in the general population is currently estimated at 8 to 10%) are associated with a higher risk of developing a range of behavioral and health disorders, which occur at much higher rates among individuals with ACEs [14,15]. The newest surveys show that alcohol, nicotine, and cannabis use are the most prevalent substances among young adults [16,17]. One possible explanation for this is that the use of the aforementioned substances is associated with attempts to manage internal stress or emotional pain [18]. This view has some support, given that individuals with higher scores on ACEs are more likely to prefer such measures [19].

Three aspects are important when investigating the association model between ACEs and SUDs. The first is that it recognizes that different risk factors can lead to similar outcomes, and that risk factors themselves are often interrelated. Therefore, individuals who have one adverse childhood experience are often exposed to others. The second is that adverse childhood experiences tend to have a dose–response relationship with a range of adverse outcomes; the greater the amount of hardship experienced, the more progressive the adverse effects on development and functioning in various domains. Thirdly, ACEs can have long-term consequences [6]. Many studies suggest that ACEs increase the likelihood of maladaptive coping strategies, including early onset of addictions and long-term psychoactive substance use [13].

Early studies have shown that those who have experienced four or more adverse childhood experiences by adulthood have a four- to twelve-fold increased risk of developing alcohol or drug use problems. Studies are usually conducted by adding up all adverse childhood experiences, with each additional ACEs increasing the likelihood of starting to use psychoactive substances by two to four times, suggesting a dose–response relationship between ACEs and substance use disorders [20]. However, adverse childhood experiences range from emotional neglect or parental imprisonment to frequent physical or sexual abuse, which of course cannot be compared and measured with the same weight. Many previous studies have linked the total number of ACEs to psychoactive substance use, using the total ACE score as an overall risk indicator. A growing body of research highlights that different types of ACEs may have different effects on the use of certain psychoactive substances [15,21,22]. This proves the relevance of investigating the relationship between separate ACEs and psychoactive substance use in order to obtain a more precise picture of these relationships with possible implications for substance use prevention and psychotherapeutic approaches.

The number of studies systematically analyzing the association of individual negative experiences with specific substance use remains relatively small, and further research is needed to examine the specific relationships between individual ACE types and different forms of substance use in detail [23]. The aim of this study is to analyze the associations of individual types of adverse childhood experiences with the prevalence of cannabis, alcohol, nicotine, and other heavy psychoactive substance use among young adults, using standard questions on negative childhood experiences, and to see which adverse childhood experiences have the strongest associations with which specific psychoactive substances.

## 2. Materials and Methods

### 2.1. Participants and Procedure

Data were collected in Lithuania from September to November 2024, using a convenience sampling method. Questionnaires were distributed without knowing whether respondents had experienced adverse events or used substances, Respondents completed the survey, hosted on Google Forms. All participants were recruited through social media and via emails sent by the research team, which requested that they forward the invitation to participate to others. We also approached students of Mykolas Romeris University and Lithuanian University of Health Sciences with a request to participate in the study. It must be noted that students who were approached with an invitation to participate in the study in-person were studying the social sciences, therefore the data are more representative of females and should be interpreted in this context. Individuals aged 18 to 29 were invited to participate; however, due to convenience sampling, 65.4% of the participants were between 18 and 22 years of age. A total of 709 individuals completed the questionnaire, comprising 188 males (26.5%) and 521 females (73.5%). Participants were informed that the survey was anonymous, and they were free to withdraw from the survey at any point if they wished to do so.

### 2.2. Measurements

#### 2.2.1. Genealogical Context and Demographic Data Collection

Participants were asked to provide information about themselves, indicating biological sex and age. To reveal genealogy, targeted questions were used together with demographic data: “Do you think/know that your father/mother was addicted to alcohol/other psychoactive substances?”; “Do you think/know that your grandfather/grandmother was addicted to alcohol/other psychoactive substances?”; “Did your father/mother smoke cigarettes?”; “Did your grandfather/grandmother smoke cigarettes?” The scale demonstrated reasonable internal consistency: KR-20 (Kuder–Richardson Formula 20) = 0.573.

For individual nicotine use we included a question, “Do you smoke?” This question is coded as D5 in the results.

#### 2.2.2. Adverse Childhood Experiences (ACEs)

A pilot study was conducted in May 2024 using the ACEs questionnaire [20]; however, feedback indicated that some items needed to be revised—the ACE questionnaire in its original form is more suited for in-person interviews and becomes confusing to respondents when administered through an online survey. Therefore, we simplified the questionnaire in hopes of obtaining more reliable data by avoiding confusion while also retaining the construct validity of the original measure. We selected only a subset of items from the original questionnaire for use in the present study. The items used from the ACE questionnaire include categorical variables: parental divorce, domestic violence, parental substance abuse, parental mental disorders or suicide attempts, and parental problems with law enforcement. These items are coded as X in the Section 3. The scale demonstrated reasonable internal consistency, with a Cronbach’s alpha (α) of 0.698.

Other negative childhood experiences—physical neglect, emotional neglect, physical abuse, emotional abuse, sexual abuse—were assessed using the MACE (Maltreatment and Abuse Chronology of Exposure) questionnaire (Teicher MH, Parigger A., 2015) [24]. This questionnaire consists of 52 statements that retrospectively assess a person’s experiences of abuse and neglect until adulthood on a 5-point Likert scale. Subscales used were as follows: (WIV) witnessing interpersonal violence (α = 0.867; Ω = 0.876) (e.g., “Saw adults living in the household push, grab, slap or throw something at your mother (stepmother, grandmother”); (SA) sexual abuse (α = 0.801; Ω = 0.830) (e.g., “Touched or fondled your body in a sexual way”, “made inappropriate sexual comments or suggestions to you”); (PPM) parental physical maltreatment (physical abuse) (α = 0.883; Ω = 0.890) (e.g., “Intentionally pushed, grabbed, shoved, slapped, pinched, punched or kicked you”); (PPN) physical neglect (α = 0.736; Ω = 0.753) (e.g., “You did not have enough to eat”); (PEN) parental emotional neglect (α = 0.734; Ω = 0.769) (e.g., “You felt that your mother or other important maternal figure was present in the household but emotionally unavailable to you for a variety of reasons like drugs, alcohol, workaholism, having an affair, heedlessly pursuing their own goals”); (PVA) parental verbal abuse (α = 0.884; Ω = 0.886) (e.g., “Swore at you, called you names, said insulting things like calling you “fat”, “ugly”, “stupid”, etc. more than a few times a year”). Participants rated all items on a five-point Likert scale from 0 (never) to 4 (very often). All scales show good internal consistency.

#### 2.2.3. The Alcohol Use Disorders Identification Test (AUDIT)

The Alcohol Use Disorders Identification Test (AUDIT) was used to assess the severity of alcohol use. The test developed by the World Health Organization (WHO) [25] is designed to assess the risk of alcohol use. The questionnaire consists of 10 questions related to alcohol use over the past year (e.g., “How many standard units of alcohol do you drink on a typical day when you drink?”) (e.g., “How often do you drink 6 or more standard units of alcohol per day?”). Items were assessed on a five-point Likert scale, the maximum possible score of the scale is 40. The test results allow us to determine risky alcohol use (8–15 points), harmful use (16–19 points), and high levels of alcohol use (20–40), while a total score can be used to indicate the level of alcohol use. The measure demonstrated good internal consistency (α = 0.855; Ω = 0.866).

#### 2.2.4. The Cannabis Use Disorders Identification Test—Revised (CUDIT-R)

The Cannabis Use Disorders Identification Test—Revised (CUDIT-R) [26] was used to assess cannabis use severity. This test consists of ten questions to assess cannabis use in the past six months. If participants indicated cannabis use in the past six months, they were asked questions assessing problematic cannabis use. It includes yes/no questions about use problems (e.g., “Have you or anyone else been injured because of your cannabis use in the past 6 months?”). Items are rated on a 5-point Likert scale (from “Never” to “Everyday or almost every day”). Scores 0–7: Low risk. No immediate action for cannabis use disorder is necessary. Scores 8–11: High-risk use. Counseling and brief intervention are recommended, as the risk is increased in this range, but the disorder does not necessarily occur. Scores 12 and above: High likelihood of cannabis use disorder. Further assessment is needed, and referral or broader intervention may be considered. The measure demonstrated excellent internal consistency (α = 0.909; Ω = 0.919).

#### 2.2.5. The Alcohol, Smoking, and Substance Involvement Screening Test (ASSIST)

The Alcohol, Smoking, and Substance Involvement Screening Test (ASSIST) was used to assess the severity of heavy psychoactive substance use. The questionnaire consists of eight questions about individual psychoactive substances, but in this study, it was modified to measure only heavy psychoactive substances as a whole, as a separate group, and the consequences arising from it, i.e., cocaine, amphetamine-type stimulants, inhalants, sedatives, hallucinogens, opioids, and new psychoactive substances. If participants indicated hard drug use in their lifetime, they were asked questions assessing problematic hard drug use in the past three months. It includes questions about use problems (e.g., “Frequency of use of each substance in the past three months”). The assessment is presented in terms of risk scores, which are divided into “low risk”, “medium risk”, or “high risk”. Low risk level (0–3 points): Low risk of health and other problems from current pattern of use. Rarely used, no visible problems—risk education recommended. Moderate (4–26): Respondent is at risk of health and other problems from current pattern of substance use. Possible problems, more frequent use—brief intervention suggested. High (27+): Respondent is at high risk of experiencing severe problems (health, social, financial, legal, relationship problems) because of his current pattern of use and are likely to be dependent. Visible manifestations of addiction or harmful use—specialist consultation and referral for treatment necessary. The measure demonstrated good internal consistency (α = 0.869; Ω = 0.876).

### 2.3. Analysis Strategy

Descriptive statistics, including linear relationships among all variables of the model, were used to assess whether the variables are appropriate for structural equation modeling. Skewness and kurtosis values were used to assess the approximate normality of the distributions of the variables.

To test hypotheses about linear relationships, we used structural equation modeling, using the robust DWLS (diagonally weighted least squares) estimation method, which is particularly suitable for analyzing ordinal variables [27]. Structural equation modeling is a useful method in such models as it allows us to estimate latent variables despite varying internal consistency measures among scales, thus providing more robust effect sizes than a simple multiple regression would. Additionally, structural equation modeling allows us not only to assess individual effects, but the model as a structure, therefore allowing at least some theory-building based on data [28,29].

We report χ^2^, CFI (comparative fit index), TLI (Tucker–Lewis index), and RMSEA (root mean square error of approximation) as indicators of model fit, and we regard CFI and TLI values of 0.95 and higher and RMSEA values of 0.07 and lower as indicative of a good model fit [30]. CFI and TLI indices reflect how well the model fits the data compared to a null model, while RMSEA measures how far away a model is from perfect. SRMR indicates how far the predicted correlations differ from observed ones, providing a good metric of model functioning. Missing values were handled by pairwise deletion. Jamovi software (version 2.6) was used for statistical analysis.

## 3. Results

### 3.1. Pathways from Childhood Adversity to Substance Use

#### 3.1.1. Descriptive Statistics for All Variables Used in the Model

Descriptive statistics for all variables used in the model are presented in Table 1. All variables were found to be interrelated with medium to large effects. As expected, all variables have acceptable skewness and kurtosis to approximate normality with the exception of sexual abuse, which does not follow a normal distribution because the relative rarity of sexual abuse results in a very steep distribution with not a lot of variance. However, we still include the variable in the structural model because our choice of estimation and the relatively large sample size allow us to obtain valuable data even with variables that are not strictly normally distributed.

#### 3.1.2. A Structural Model Predicting Psychoactive Substance Use

A structural model predicting psychoactive substance use was constructed and tested (see Table 2). The model demonstrated good fit to the data (RMSEA = 0.077 [0.075, 0.079], pclose < 0.001; CFI = 0.974; TLI = 0.971; χ^2^(1018) = 5249, *p* < 0.001. R2AUDIT = 0.327; R2CUDIT = 0.185; R2ASSIST = 0.546; R2D5 = 0.147) (see Table A1). For alcohol use (AUDIT), significant predictors were parental verbal abuse (which, paradoxically, has a negative effect, meaning that those who experienced more verbal abuse were less likely to abuse alcohol in adulthood), physical maltreatment, and sexual abuse, which are all correlated positively with alcohol use. For cannabis use (CUDIT), only sexual abuse was found to be a significant predictor, but an effect approaching statistical significance is observed for parental verbal abuse, with the effect being in the same direction as alcohol abuse. Heavy psychoactive substance use (ASSIST) was, again, negatively predicted by parental verbal abuse and positively by sexual abuse, with a negative relationship with the absence of parental emotional neglect which was approaching statistical significance.

Nicotine use (reported by participants, coded as D5 in Table 2) is significantly predicted by participants’ reported sexual abuse and their witnessing of interpersonal violence in the family.

## 4. Discussion

There has already been a lot of research in the scientific community that shows quite clear links between adverse childhood experiences and the use of psychoactive substances in adulthood. About 90 studies, from the comprehensive systematic review conducted by Sebalo and colleagues in 2023, confirmed a clear link between various adverse childhood experiences and a higher risk of using alcohol, cannabis, and other psychoactive substances in young adults. However, this topic is still new and research is ongoing [31,32,33,34,35].

Our study aimed to analyze the associations of different ACE types with the prevalence of cannabis, alcohol, nicotine, and other heavy psychoactive substance use among young adults using the standard ACE questionnaire (10 negative childhood experiences). The results of our study lead to a better understanding of the links between ACEs and substance use in young adulthood: only sexual abuse is associated with higher severity of psychoactive substance use across multiple substance categories, and only alcohol showed a relationship with physical violence, and nicotine showed a relationship with witnessing interpersonal violence.

Research suggests that there is a relationship between family history of addiction and psychoactive substance use, i.e., a genealogical component [36,37,38]. No significant associations between family history of addiction and psychoactive substance use were observed in our study. However, limitations in self-reported data and sample characteristics preclude firm conclusions regarding heritability. However, this could highlight the potential dominance of environmental and psychosocial factors. Indeed, this may have been influenced by at least a few factors, such as the young age of the respondent (65.4% of the participants were 18 to 22 years of age) and the fact that addictions together with other mental disorders usually occur before the brain system is fully mature, i.e., up to 25 years [39], so it may be that the participants in the study were too young. There is also a cultural aspect, which points to addictions/harmful use being common in Eastern Europe; this could explain why the third generation may not be able to correctly identify the history of addictions in their family members, simply not remember them, or even unconsciously use defense mechanisms of repression when remembering.

Among the ten adverse childhood experiences examined, different types of experiences may exert varying influences on substance use. In our study, one of the family dysfunction factors (witnessing interpersonal violence) was associated only with nicotine use. Further research is needed to compare the influence of individual adverse childhood experiences on the use of psychoactive substances, especially distinguishing the latent variable of family dysfunction. Previous studies have suggested that personal experiences of maltreatment may have particularly detrimental effects on later mental and physical health outcomes [40]. Experiencing four or more adverse childhood experiences generally indicates a high risk in adulthood for both mental disorders, including addictions, and physical illnesses, such as cancer or autoimmune diseases [20,40].

But it would not be fair to compare between some cases, for example, if four or more experiences are about family dysfunction, and in another case, four or more experiences are about internal experiences including sexual violence. Therefore, the study may consider excluding ACEs from the overall summation and instead identify the most influential adverse experiences and measure them separately.

In our study, only sexual abuse was associated with the cannabis use. Other authors also noticed that sexual abuse is most associated with higher odds of cannabis use [41]. At the same time, some studies show that household mental illness and parental substance use are also high-risk factors; furthermore, the prevalence of cannabis use increases with ACE count [42].

We found that nicotine and heavy psychoactive substance use (ASSIST) in adulthood is associated with sexual abuse and the witnessing of interpersonal violence in the family in childhood, similarly to other authors [43]. Furthermore, heavy psychoactive substance use was negatively associated with parental verbal abuse and positively associated with sexual abuse, with a negative relationship with the absence of parental emotional neglect which approached significance. The data from other studies show that supportive parenting is associated with lower rates of drug use among adolescents [44,45,46]. On the other hand, after a comprehensive assessment, other authors noted that emotional abuse and neglect may influence adolescent substance use. Yet their effects are often weaker or more indirect compared to other maltreatment types [47]. Such varied results suggest that the absence of parental emotional neglect in childhood is not such a strong predictor for heavy substance use in adulthood.

A large amount of scientific data shows that verbal/emotional abuse in childhood is associated with higher odds of alcohol and heavy psychoactive substance use disorders in adulthood [35,48,49]. In contrast, we found that parental verbal abuse was negatively associated with substance use in several domains (cannabis, heavy psychoactive substances and alcohol). There are also studies that have found that alcohol use in adulthood is determined solely by child neglect and emotional abuse [50,51]. In our study, we found the opposite result—verbal abuse experienced in childhood was associated with a lower risk of alcohol abuse in adulthood. Several factors could have contributed to these differences—the first, sampling characteristics. For instance, it could be due to familial norms, whereby parenting verbal abuse that includes insulting or humiliating a child, etc. is more related to the authoritarian parenting style, which is also characterized by strict control over the child, and in studies, it shows lower associations with the use of psychoactive substances than the permissive or uninvolved parenting style [52,53,54,55]. At the same time, if our study groups were made from subjects from families with strong anti-alcohol norms, adversity might increase the risk of other problems (depression, anxiety) without translating into alcohol misuse. Furthermore, the majority of the study participants were women. Due to hormonal differences and differences in HPA axis activity between the sexes, women’s responses to verbal abuse may also differ when compared to studies of the general population [56,57]. Secondly, psychological mechanisms. It may be that the individuals in our sample who experienced verbal abuse became particularly cautious and actively avoided alcohol consumption to protect themselves, or developed strong self-control or control tendencies that made them less likely to engage in risky behavior, such as drinking. It may also be that the subjects chose intense involvement in games as a way of coping with the stress they experienced (verbal abuse) instead of alcohol, which means that our sample was more inclined toward internalizing (e.g., games, social media) rather than classic externalizing outcomes (alcohol or drugs). Summarizing, this finding could be explained by the fact that strict parenting reduces the risk of using psychoactive substances in adulthood due to acquired discipline and strong self-regulatory mechanisms. And the third one—methodical and statistical differences between studies. What we might be observing in our data is that while all forms abuse likely include a verbal component, verbal abuse on its own, when all other abuse is controlled for in the model, paradoxically reverses its effect, reflecting not the fact that verbal abuse leads to desired outcomes, but that its unique variance, when all other abuse is accounted for in the model, tends to associate with the outcome variable negatively because it reflects more so the absence of other forms of abuse rather than verbal abuse itself. Still, further research is needed to compare the influence of adverse childhood experiences and parenting styles on the use of psychoactive substances and to investigate this curious finding further.

Perhaps the bigger problem in the Western world today is parenting styles that do not develop a child’s resilience; this makes them hypersensitive to experiences, interpreting them as traumatic, although the real cause is permissive and uninvolved parenting styles.

As with all studies, the present study has some limitations. The main objective of this study was to review the associations between individual adverse childhood experiences and individual psychoactive substances, which is why the covariates, such as biological sex, resilience, and social support, were not included in the structural equation modeling; the model does not converge when they are included. The sample, although large and sufficient for the model, is not representative. Our study primarily consisted of students, most of whom were women. Given that the stress/HPA response profile differs between the sexes, we can conclude that the results of our study reflect those of the population of young women with higher education. Social desirability could have had a significant impact on quantitative study, especially when asking about such sensitive experiences in childhood and the use of psychoactive substances [58]. Thus, the summary of the results should be evaluated cautiously. Generational differences should also be taken into account. We have singled out young adults, but this term does not distinguish between the generational differences themselves, which are obviously enormous in the digital age; therefore, future research could focus more on examining differences between generations rather than between different age groups. Still, a deeper analysis is needed to clarify this phenomenon. Furthermore, the assessment of substance use in this study focused on the frequency and presence of use, without the means to distinguish between non-problematic and abusive patterns, which should be addressed in future research.

The insights of our study emphasize the critical need for trauma-informed prevention and intervention strategies targeting young adults exposed to early adversity. Thus, established results not only brought knowledge to science, but also are useful in practice in terms of preventing the development of addictions, since the addiction syndrome most often develops in young adults.

## 5. Conclusions

The main results of this study showed that adverse childhood experiences (ACEs) are not qualitatively equivalent to one another, so it is worth examining them separately, rather than summing them. Also, from the negative associations with verbal abuse and the largely statistically negative associations, we can predict that ACEs are not the most influential, so further studies should investigate other factors.

## Figures and Tables

**Table 1:** Descriptive statistics of all used variables.

	Mean	SD	S	K	1	2	3	4	5	6	7
1. Parental Verbal Abuse	1.0730	1.092	0.960	4.46 × 10^−4^	—						
2. Absence of Parental Emotional Neglect	2.9587	1.018	−0.851	−0.0486	−0.568 ***	—					
3. Absence of Parental Physical Neglect	3.3929	0.736	−1.395	1.2557	−0.404 ***	0.706 ***	—				
4. Physical Abuse	0.5790	0.780	1.959	3.8465	0.678 ***	−0.438 ***	−0.377 ***	—			
5. Sexual Abuse	0.0889	0.319	6.341	52.7672	0.310 ***	−0.291 ***	−0.341 ***	0.391 ***	—		
6. Witnessing Interpersonal Violence	0.3337	0.680	2.595	6.8113	0.479 ***	−0.343 ***	−0.298 ***	0.473 ***	0.387 ***	—	
7. Family History of Addiction	1.8577	1.817	0.742	−0.3576	0.456 ***	−0.355 ***	−0.246 ***	0.322 ***	0.170 ***	0.432 ***	—
8. Dysfunctional Family	2.2976	1.281	−0.218	−0.9877	0.311 ***	−0.221 ***	−0.150 ***	0.202 ***	0.083 *	0.268 ***	0.466 ***

Note. * *p* < 0.05, *** *p* < 0.001.

**Table 2:** Results of the tested model.

				95% Confidence Intervals				
Dep	Pred	Estimate	SE	Lower	Upper	β	z	*p*
AUDIT	Parental verbal abuse	−3.0398	1.1519	−5.2975	−0.782	−0.48742	−2.6389	0.008
AUDIT	Absence of parental emotional neglect	−3.9640	3.0738	−9.9885	2.061	−0.53795	−1.2896	0.197
AUDIT	Absence of physical neglect	3.7788	2.4657	−1.0540	8.612	0.61461	1.5325	0.125
AUDIT	Physical maltreatment	2.1308	0.7184	0.7227	3.539	0.34656	2.9659	0.003
AUDIT	Sexual abuse	3.6905	0.5077	2.6954	4.686	0.63552	7.2692	<.001
AUDIT	Witnessing interpersonal violence	−0.4041	0.4635	−1.3125	0.504	−0.06753	−0.8719	0.383
AUDIT	Dysfunctional family	0.0457	0.9523	−1.8208	1.912	0.00773	0.0480	0.962
AUDIT	Family history of addiction	1.9009	2.4599	−2.9204	6.722	0.13238	0.7728	0.440
CUDIT	Parental verbal abuse	−5.2896	3.1411	−11.4460	0.867	−0.58996	−1.6840	0.092
CUDIT	Absence of parental emotional neglect	−10.5634	8.0775	−26.3951	5.268	−0.99713	−1.3077	0.191
CUDIT	Absence of physical neglect	7.9404	6.4374	−4.6766	20.557	0.89833	1.2335	0.217
CUDIT	Physical maltreatment	2.4625	2.0001	−1.4577	6.383	0.27858	1.2312	0.218
CUDIT	Sexual abuse	3.5237	1.3814	0.8162	6.231	0.42207	2.5508	0.011
CUDIT	Witnessing interpersonal violence	−0.0887	1.3224	−2.6805	2.503	−0.01031	−0.0671	0.947
CUDIT	Dysfunctional family	0.6064	2.5897	−4.4694	5.682	0.07126	0.2341	0.815
CUDIT	Family history of addiction	−0.9410	6.7083	−14.0891	12.207	−0.04558	−0.1403	0.888
ASSIST	Parental verbal abuse	−8.2574	3.9382	−15.9761	−0.539	−0.77884	−2.0967	0.036
ASSIST	Absence of parental emotional neglect	−19.4373	10.5767	−40.1672	1.293	−1.55165	−1.8378	0.066
ASSIST	Absence of physical neglect	13.1400	8.4191	−3.3612	29.641	1.25716	1.5607	0.119
ASSIST	Physical maltreatment	2.7379	2.4842	−2.1310	7.607	0.26194	1.1021	0.270
ASSIST	Sexual abuse	6.8392	1.7161	3.4756	10.203	0.69279	3.9852	<.001
ASSIST	Witnessing interpersonal violence	0.8736	1.5650	−2.1937	3.941	0.08587	0.5582	0.577
ASSIST	Dysfunctional family	3.4613	3.2966	−3.0000	9.923	0.34402	1.0499	0.294
ASSIST	Family history of addiction	−9.5849	8.4209	−26.0895	6.920	−0.39263	−1.1382	0.255
D5	Parental verbal abuse	−0.0304	0.2167	−0.4550	0.394	−0.02794	−0.1401	0.889
D5	Absence of parental emotional neglect	−0.2438	0.5884	−1.3972	0.910	−0.18992	−0.4144	0.679
D5	Absence of physical neglect	0.3135	0.4724	−0.6125	1.239	0.29262	0.6635	0.507
D5	Physical maltreatment	0.2028	0.1435	−0.0784	0.484	0.18936	1.4134	0.158
D5	Sexual abuse	0.4012	0.1055	0.1944	0.608	0.39652	3.8027	<.001
D5	Witnessing interpersonal violence	−0.3223	0.0954	−0.5093	−0.135	−0.30908	−3.3776	<.001
D5	Dysfunctional family	0.0595	0.1725	−0.2786	0.398	0.05775	0.3452	0.730
D5	Family history of addiction	0.2187	0.4518	−0.6668	1.104	0.08741	0.4841	0.628

## Data Availability

The data supporting the findings of this study are available from the corresponding author upon reasonable request.

## Abbreviations

The following abbreviations are used in this manuscript:

ACEs	Adverse childhood experiences
SUD	Substance use disorders

## Appendix A

**Table A1:** Measurement model.

				95% Confidence Intervals				
Latent	Observed	Estimate	SE	Lower	Upper	β	z	*p*
Parental verbal abuse	PVA1	1.000	0.0000	1.000	1.000	0.920		
	PVA2	0.974	0.0161	0.943	1.006	0.897	60.4	<0.001
	PVA3	0.998	0.0148	0.969	1.027	0.919	67.3	<0.001
	PVA4	0.894	0.0149	0.864	0.923	0.822	60.1	<0.001
Parental emotional neglect	PEN1R	1.000	0.0000	1.000	1.000	0.779		
	PEN2R	−0.916	0.0220	−0.959	−0.873	−0.713	−41.6	<0.001
	PEN3	1.201	0.0208	1.161	1.242	0.936	57.8	<0.001
	PEN4	1.200	0.0208	1.159	1.240	0.934	57.7	<0.001
	PEN5	1.140	0.0210	1.099	1.181	0.888	54.3	<0.001
Parental physical neglect	PPN1	1.000	0.0000	1.000	1.000	0.934		
	PPN2	0.913	0.0148	0.884	0.942	0.852	61.6	<0.001
	PPN3R	0.697	0.0183	0.661	0.732	0.650	38.1	<0.001
	PPN4R	−0.817	0.0207	−0.857	−0.776	−0.762	−39.4	<0.001
	PPN5	0.916	0.0126	0.891	0.941	0.855	72.5	<0.001
Parental physical maltreatment	PPM1	1.000	0.0000	1.000	1.000	0.934		
	PPM2	0.991	0.0164	0.959	1.024	0.925	60.3	<0.001
	PPM3	1.010	0.0199	0.971	1.049	0.943	50.6	<0.001
	PPM4	0.933	0.0143	0.905	0.961	0.871	65.4	<0.001
	PPM5	0.848	0.0165	0.816	0.880	0.791	51.4	<0.001
	PPM6	0.931	0.0142	0.903	0.959	0.869	65.7	<0.001
Sexual abuse	SA1	1.000	0.0000	1.000	1.000	0.988		
	SA2	0.926	0.0266	0.874	0.979	0.916	34.8	<0.001
	SA3	1.000	0.0270	0.947	1.053	0.988	37.1	<0.001
	SA4	0.932	0.0269	0.880	0.985	0.922	34.7	<0.001
	SA5	0.770	0.0270	0.717	0.823	0.761	28.5	<0.001
	SA6	0.967	0.0254	0.917	1.017	0.956	38.1	<0.001
	SA7	0.962	0.0254	0.912	1.012	0.951	37.9	<0.001
Witnessing interpersonal violence	WIV1	1.000	0.0000	1.000	1.000	0.959		
	WIV2	1.017	0.0203	0.978	1.057	0.976	50.0	<0.001
	WIV3	0.897	0.0180	0.862	0.933	0.861	49.7	<0.001
	WIV4	0.896	0.0170	0.863	0.930	0.860	52.7	<0.001
	WIV5	0.911	0.0188	0.874	0.948	0.874	48.5	<0.001
Family addiction history	D1	1.000	0.0000	1.000	1.000	0.970		
	D2	0.647	0.0268	0.595	0.700	0.627	24.1	<0.001
	D3	0.492	0.0270	0.439	0.545	0.477	18.3	<0.001
	D4	0.441	0.0256	0.391	0.491	0.428	17.2	<0.001
Household dysfunction	X1	1.000	0.0000	1.000	1.000	0.400		
	X2	0.901	0.0843	0.735	1.066	0.360	10.7	<0.001
	X3	1.657	0.1041	1.453	1.861	0.662	15.9	<0.001
	X4	1.759	0.1026	1.558	1.960	0.703	17.1	<0.001
	X5	1.788	0.1015	1.589	1.987	0.715	17.6	<0.001
	X6	1.744	0.1011	1.546	1.943	0.697	17.2	<0.001
	X7	2.443	0.1246	2.199	2.688	0.977	19.6	<0.001

Initial value.
